# Morphology and Morphometric Analysis of the Psoas Minor Muscle in Adult Human Cadavers at a Tertiary-Care Institution in Western India

**DOI:** 10.7759/cureus.111450

**Published:** 2026-06-24

**Authors:** Bhoopesh Raja, Manoj M Kulkarni, Hetal Vaishnani

**Affiliations:** 1 Anatomy, Smt. B. K. Shah Medical Institute and Research Centre, Sumandeep Vidyapeeth Deemed to Be University, Vadodara, IND

**Keywords:** anatomical variation, cadaveric dissection, iliopubic eminence, lumbar plexus, morphometry, posterior abdominal wall, psoas minor, vestigial muscle, western indian population

## Abstract

Introduction: The psoas minor is a slender, inconstantly present vestigial muscle of the posterior abdominal wall. Classical texts report its presence in 40-50% of individuals. Still, considerable ethnic and geographic variation has been documented, and morphometric data from Western Indian cadaveric populations remain scarce despite their growing relevance to retroperitoneal surgery, abdominopelvic imaging, and lumbopelvic physiotherapy. Aim: This study aims to determine the observed frequency and document the morphology and morphometric characteristics of the psoas minor muscle in adult human cadavers available for routine dissection at a single tertiary-care institution in Vadodara, Gujarat (Western India). Materials and methods: This cross-sectional observational cadaveric study was conducted in the Department of Anatomy, Smt. B.K. Shah Medical Institute and Research Centre, Sumandeep Vidyapeeth, Vadodara, on cadavers dissected during routine undergraduate teaching between January 2022 and January 2026; institutional ethics committee approval was obtained for the retrospective analysis and reporting of these pre-existing dissection findings. Thirty adult formalin-embalmed cadavers (60 sides) allocated for routine undergraduate dissection across three consecutive MBBS batches were systematically examined. The posterior abdominal wall was exposed, and the psoas major was reflected medially to inspect for the psoas minor. When identified, origin, insertion, number of bellies, belly length, tendon length, total length, and maximum belly diameter were recorded using a digital caliper and non-elastic thread. Descriptive statistics were computed using SPSS Statistics version 26.0 (IBM Corp. Released 2019. IBM SPSS Statistics for Windows, Version 26.0. Armonk, NY: IBM Corp.).

Results: The psoas minor was identified in two of 30 cadavers (6.67%; exact binomial 95% CI 0.8-22.1%), corresponding to three of 60 sides (5.00%; 95% CI 1.0-13.9%). One cadaver exhibited a bilateral psoas minor, and one showed a unilateral left-sided muscle. All three muscles originated from the lateral aspect of the bodies of T12 and L1 and inserted, via a long, slender tendon, into the pecten pubis and iliopubic eminence, with the tendon also blending laterally with the iliac fascia. The mean belly length was 4.57 ± 0.46 cm, the mean tendon length was 17.17 ± 0.85 cm, the mean total length was 21.73 ± 0.45 cm, and the mean belly diameter was 0.60 ± 0.05 cm.

Conclusions: The observed frequency of the psoas minor in this single-institution cadaveric sample from Vadodara, Gujarat (6.67%) is considerably lower than the classically reported prevalence of 40-50%, consistent with growing evidence of regional and ethnic variation. Awareness of its infrequent presence and morphometric profile is clinically relevant during retroperitoneal surgery, radiological interpretation, psoas compartment block, and physiotherapeutic management of lumbopelvic dysfunction.

## Introduction

The posterior abdominal wall is anatomically complex, housing major vascular structures such as the abdominal aorta, inferior vena cava, common iliac vessels, and the azygos system, as well as the lumbar plexus and its terminal branches, including the iliohypogastric, ilioinguinal, genitofemoral, lateral femoral cutaneous, femoral, and obturator nerves. This wall is predominantly supplied by the quadratus lumborum, iliacus, and the psoas muscle group, the last of which comprises the psoas major and psoas minor [1,2]. Among these, the psoas minor is the most inconsistently present component and is widely regarded as a vestigial structure in humans [3,4].

Classically, the psoas minor arises from the lateral surface of the bodies of the twelfth thoracic and first lumbar vertebrae and the intervening intervertebral disc; it lies entirely within the abdomen along the anterior aspect of the psoas major; and it ends in a long, flattened tendon that inserts into the pecten pubis, iliopubic eminence, and laterally into the iliac fascia [1,3,5]. Functionally, it is described as a weak flexor of the lumbar spine and a tensor of the iliac fascia. Still, its contribution is considered clinically trivial owing to its small bulk and variable presence [2,6]. Nerve supply is from the first lumbar spinal nerve (L1) through the lumbar plexus [3,5]. The muscle may present unilaterally or bilaterally and, occasionally, with a double belly [6,7].

The reported prevalence of the psoas minor in the human population shows striking inter-ethnic and inter-regional variability. Standard textbooks cite a prevalence of approximately 40-50% [1,3,4], whereas independent cadaveric studies report figures ranging from as low as 8% to as high as 90% [7-11]. Hanson et al. noted anatomical differences between individuals of European and African descent, with the psoas minor present more frequently in the former [12]. Patil et al., in a North Indian cohort, observed a moderately high reported frequency with considerable morphometric variability [9]. Conversely, Kokati et al., in a South Indian cadaveric series, reported a comparatively lower frequency and emphasized a likely ethnic influence [10]. Gupta et al. recently published a case report highlighting a rare bilateral presentation and its embryological and evolutionary significance [13]. Karrar further documented an atypical insertion of the psoas minor tendon into the greater trochanter in a 52-year-old Saudi Arabian female cadaver [14].

Beyond anatomical interest, the psoas minor carries notable clinical and surgical implications. Its tendon is intimately related to the femoral nerve and the psoas sheath, making it relevant during psoas compartment blocks, retroperitoneal lymph node dissection, anterior lumbar interbody fusion, living-donor nephrectomy, and urological retroperitoneoscopic procedures [15,16]. On imaging, awareness of the variable morphology and asymmetry of the psoas minor is important when evaluating the retroperitoneum on computed tomography (CT) and magnetic resonance imaging (MRI), as a prominent or unilaterally hypertrophic muscle could otherwise be misinterpreted [17,18]. Physiotherapists treating chronic low back pain, anterior pelvic tilt, and iliopsoas-related disorders may also benefit from awareness of its variable presence [19]. Bilateral persistence and other atypical presentations of the muscle have been documented in cadaveric series and should be carefully recorded during dissection [20].

Gujarat-specific cadaveric data on the frequency and morphometry of the psoas minor are sparse. The present observational cadaveric study was therefore undertaken to document the observed frequency, morphology, and morphometric profile of the psoas minor muscle in adult human cadavers available for routine undergraduate dissection at a single tertiary-care medical institution in Vadodara, Gujarat (Western India), and to compare these findings with earlier reports from other populations.

## Materials and methods

Study design and setting

This observational cross-sectional cadaveric study is based on dissections performed in the Department of Anatomy, Smt. B.K. Shah Medical Institute and Research Centre (SBKS MI&RC), Sumandeep Vidyapeeth, Deemed to be a University, Piparia, Vadodara, during routine first-year MBBS teaching between January 2022 and January 2026. These cadaveric observations were made in the course of routine undergraduate dissection, which did not itself require prospective ethical approval; institutional ethics committee approval was subsequently obtained for the retrospective compilation, analysis, and reporting of these pre-existing dissection findings (Sumandeep Vidyapeeth Institutional Ethics Committee; reference no. SVIEC/ON/Med/SRP1/Feb/26/124, dated 25 February 2026). All cadavers were legally willed, formalin-embalmed adult specimens allocated to three consecutive first-year MBBS batches for routine dissection, and no living participants were involved. The study adhered to the Declaration of Helsinki (2013 revision) and institutional guidelines governing cadaveric research and donor confidentiality. Standardized protocols and calibrated measurement tools were used throughout.

Sample size and sampling

All eligible adult human cadavers available for routine undergraduate dissection during the study period were included by complete enumeration (consecutive sampling), giving a total of 30 cadavers (60 sides, 30 right and 30 left). No formal sample-size calculation was performed; the study is descriptive, and the sample reflects complete enumeration of the cadavers available during the study period rather than a statistically powered sample. Because the psoas minor is an inconsistently present structure, this number of cadavers limits the precision of the frequency estimate, and the morphometric findings should be interpreted with this constraint in mind [7,9,10].

Inclusion and exclusion criteria

Adult human cadavers of either sex (≥18 years) allocated for routine undergraduate dissection during the study period were included, provided that the posterior abdominal wall and psoas compartment were grossly intact bilaterally and that the vertebral column, psoas major, iliacus, lumbar plexus, and iliopubic region were clearly identifiable on both sides. Cadavers were excluded if there was evidence of previous abdominal or spinal surgery, trauma, tumor, or congenital anomaly involving the posterior abdominal wall, or if the posterior abdominal musculature was mutilated, lacerated, or decomposed to an extent that rendered the origin or insertion of the psoas compartment unidentifiable.

Dissection technique

Each cadaver was placed supine on the dissection table. The anterior abdominal wall was opened by a combined midline and bilateral costal-pubic incision and reflected laterally. The abdominal viscera were reflected, and the abdominal aorta, inferior vena cava, and lumbar plexus branches were traced and preserved. The parietal peritoneum, extraperitoneal fat, and fascia transversalis overlying the posterior abdominal wall were meticulously cleared to reveal the quadratus lumborum, iliacus, and psoas major. The psoas major was gently retracted medially along its lateral border to inspect the lateral aspect of the T12 and L1 vertebral bodies for any slender, fusiform muscle representing the psoas minor. When present, the muscle was cleaned of fascial remnants and traced along its entire course from origin to insertion on the pecten pubis and iliopubic eminence. Its identity was confirmed by its characteristic position immediately anterior to the psoas major, its origin from the T12-L1 region, and its long, flat tendon descending towards the pecten pubis and iliopubic eminence.

Parameters observed

The following parameters were documented independently for the right and left sides of each cadaver: presence or absence of the psoas minor; pattern of occurrence (unilateral right, unilateral left, or bilateral); vertebral level of origin; site of insertion; number of bellies (single or double); length of the muscular belly in centimetres, measured from the origin to the musculotendinous junction; length of the tendon in centimetres, measured from the musculotendinous junction to the insertion; total length of the muscle in centimetres, calculated as the sum of belly and tendon lengths; and maximum diameter of the belly in centimetres, measured at the widest point perpendicular to the long axis of the muscle.

Linear measurements were obtained in situ, before removal of the muscle, with a stainless-steel digital vernier caliper (measuring range 0-150 mm; resolution 0.01 mm) and a non-elastic thread laid along the central axis of the tendon and then measured against a metric scale to accommodate its curved course. The musculotendinous junction, used as the landmark for the belly and tendon lengths, was identified macroscopically, and the caliper was checked against its zero reading before each measurement session. Each measurement was recorded twice by the primary investigator and independently verified by the guide, and the mean of the two readings was used. Any morphological variation (double belly, abnormal origin, aberrant insertion) was documented photographically with a high-resolution digital camera against a metric scale.

Statistical analysis

Data were entered in Microsoft Excel 2019 (Microsoft Corp., Redmond, WA, USA) and analyzed using SPSS Statistics version 26.0 (IBM Corp. Released 2019. IBM SPSS Statistics for Windows, Version 26.0. Armonk, NY: IBM Corp.). Categorical variables were expressed as frequencies and percentages (n (%)), and continuous variables as mean ± standard deviation (SD) with minimum and maximum values. Because the psoas minor was identified in only three of 60 sides (n = 3 muscles), only descriptive statistics were used; exact (Clopper-Pearson) binomial 95% confidence intervals were calculated for the cadaver-based and side-based frequencies. No inferential statistical testing (such as Student’s t-test, chi-square test, Fisher’s exact test, or analysis of variance) was performed, as such testing would have been statistically meaningless with so few positive cases; accordingly, no significance threshold was defined, and no test statistic or p-value is reported.

## Results

A total of 30 formalin-embalmed adult human cadavers, yielding 60 sides (30 right and 30 left), were examined over the study period. Of these, 24 (80.00%) were male, and six (20.00%) were female. The baseline characteristics of the study cadavers are summarized in Table 1.

**Table 1:** Baseline characteristics of the study cadavers (n = 30)

Variable	Category	Frequency, n (%)
Sex	Male	24 (80.00)
	Female	6 (20.00)
Side examined	Right/left (paired in each cadaver)	30 (50.00)/30 (50.00)

Data are expressed as frequency n (%). Sex percentages are calculated out of the total of 30 cadavers, whereas the side counts are expressed out of the 60 sides examined (each cadaver contributing one right and one left side). No inferential statistical testing was performed on these baseline characteristics.

Frequency of the psoas minor muscle

On systematic dissection of 60 sides, the psoas minor muscle was identified in three sides (3/60; 5.00%), belonging to two cadavers (2/30; 6.67%). The overall cadaver-based frequency was therefore 6.67% (2/30; exact binomial 95% CI 0.8-22.1%), and the side-based frequency was 5.00% (3/60; 95% CI 1.0-13.9%). In one cadaver, the psoas minor was present bilaterally (right and left sides), and in a second cadaver, it was present unilaterally on the left side only; no cadaver showed an isolated right-sided muscle. In the remaining 28 cadavers (93.33%), the psoas minor was absent on both sides. The side-wise pattern of occurrence is presented in Table 2.

**Table 2:** Frequency and pattern of occurrence of the psoas minor muscle (n = 30 cadavers; 60 sides)

Pattern of occurrence	n (%)
Cadaver-level (denominator: 30 cadavers)	
Psoas minor absent bilaterally	28 (93.33)
Psoas minor present bilaterally	1 (3.33)
Psoas minor present unilaterally - left side only	1 (3.33)
Psoas minor present unilaterally - right side only	0 (0.00)
Side-level (denominator: 60 sides)	
Psoas minor present	3 (5.00)
Psoas minor absent	57 (95.00)

Data are expressed as frequency n (%). Cadaver-level patterns are calculated from 30 cadavers, and side-level frequencies from 60 sides; the two are presented separately to avoid mixing cadaver-based and side-based denominators. The overall cadaver-based frequency of the psoas minor was two of 30 cadavers (6.67%), and the overall side-based frequency was three of 60 sides (5.00%). Because the muscle was identified in only three of 60 sides, only descriptive statistics are reported, and no inferential statistical comparison (e.g., chi-square or Fisher’s exact test for side-to-side or sex-based association) was performed.

Morphology of the identified muscles

**Figure 1:**
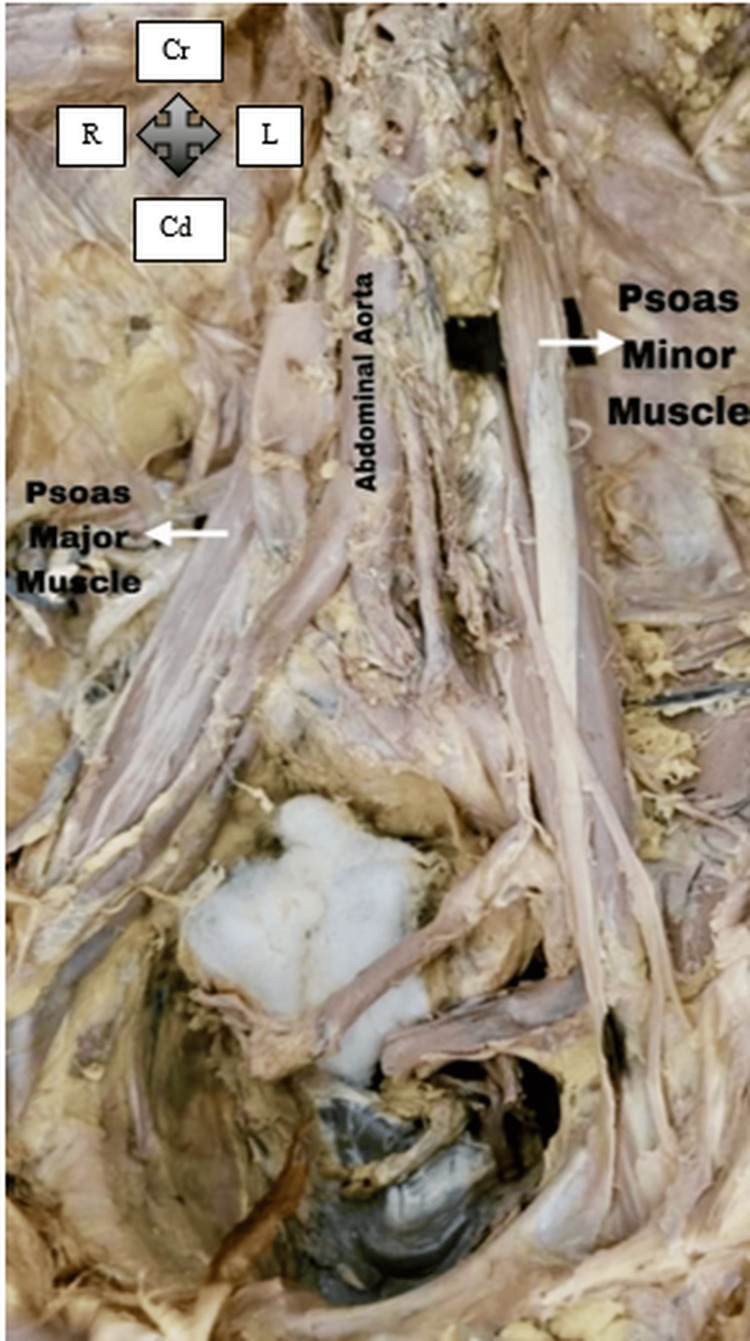
Cadaveric photograph showing a unilateral psoas minor muscle on the left side of the posterior abdominal wall R: right side, L: left side, Cr: cranial end, Cd: caudal end

**Figure 2:**
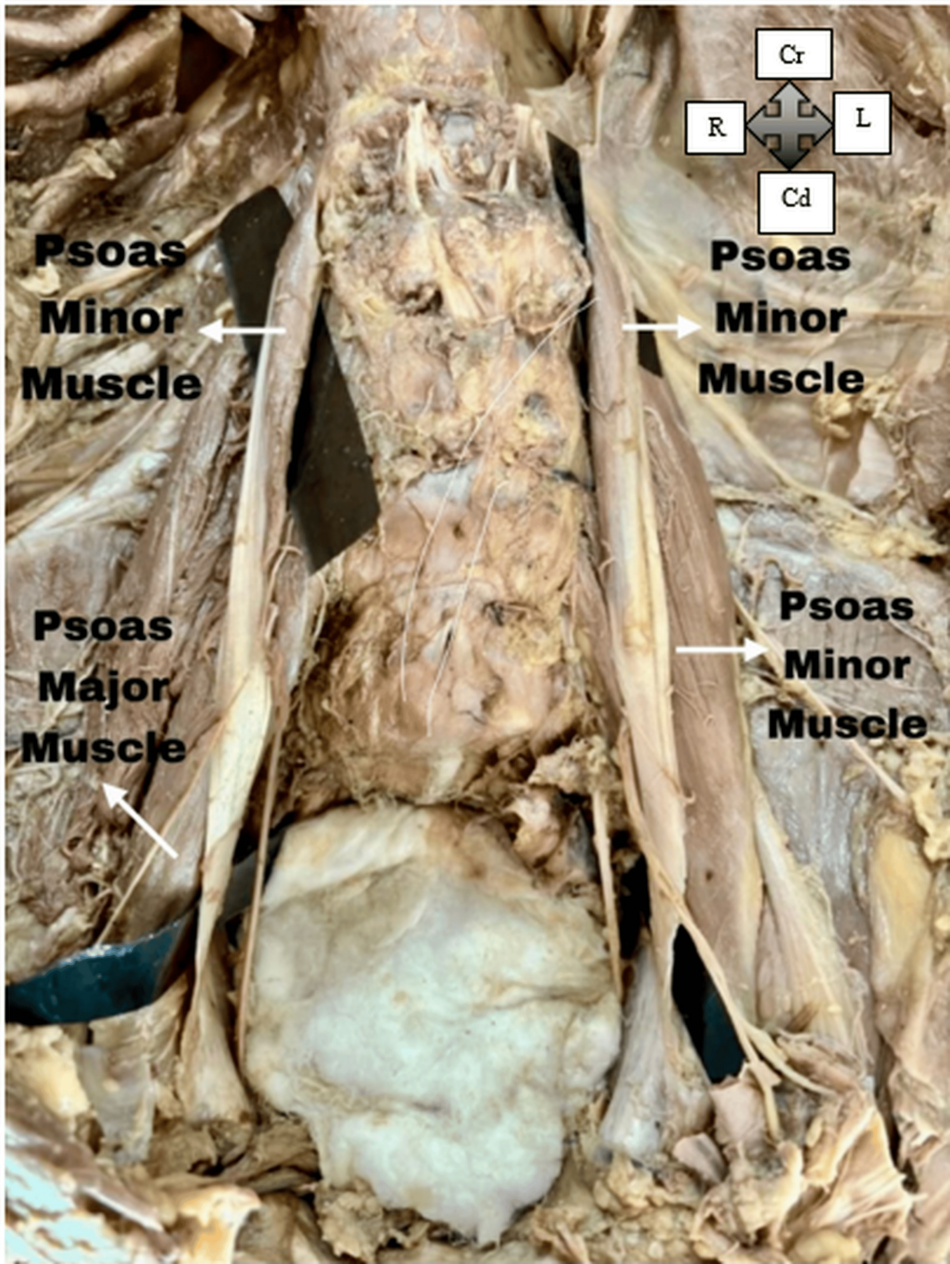
Cadaveric photograph showing a bilateral psoas minor muscle R: right side, L: left side, Cr: cranial end, Cd: caudal end

All three psoas minor muscles encountered in the present study demonstrated a classical morphological pattern. Each muscle was single-bellied, slender, and fusiform, arising from the lateral aspect of the body of the twelfth thoracic vertebra (T12) and the first lumbar vertebra (L1), together with the intervening intervertebral disc. The short, muscular belly lay immediately anterior and slightly medial to the psoas major. It continued inferiorly as a remarkably long, flattened tendon that coursed along the anterior surface of the psoas major. The tendon subsequently inserted into the pecten pubis and the iliopubic eminence, and laterally it blended with the iliac fascia covering the iliacus. None of the three muscles showed a double belly, an abnormal origin from additional vertebral levels, or an aberrant trochanteric insertion, as previously reported [14]. The representative unilateral and bilateral presentations are shown in Figure 1 and Figure 2, respectively.

The slender, pale muscle belly is seen arising from the lateral aspect of the T12-L1 vertebral region and continuing downward as a long, flattened tendon anterior to the psoas major, directed toward the pecten pubis and iliopubic eminence.

The vertebral column is shown centrally, with the paired slender psoas minor muscles (black arrow markers) lying anterior to the psoas major on both the right and left sides. The muscles arise from T12-L1 and descend as long tendons toward the pecten pubis and iliopubic eminence bilaterally.

Morphometric observations

Detailed morphometric measurements of the three identified psoas minor muscles are summarized in Table 3. The belly length ranged from 4.20 cm to 5.10 cm (mean 4.57 ± 0.46 cm), the tendon length from 16.20 cm to 17.80 cm (mean 17.17 ± 0.85 cm), and the total muscle length from 21.30 cm to 22.20 cm (mean 21.73 ± 0.45 cm). The maximum belly diameter varied narrowly between 0.55 cm and 0.65 cm (mean 0.60 ± 0.05 cm). The tendon contributed, on average, 79.00% of the total muscle length, consistent with the classical description of a small muscular belly with a disproportionately long tendon [1,3,5]. No side-to-side asymmetry exceeding 5.00% in total length was observed in the bilateral cadaver. Because only three muscles were available, these morphometric values, particularly the pooled mean ± SD, should be interpreted with caution, and the individual raw measurements presented in Table 3 are more informative than the summary statistics.

**Table 3:** Morphometric measurements of the psoas minor muscles identified in the present study (all values in cm) SD: standard deviation

Cadaver/side	Belly length	Tendon length	Total length	Max. belly diameter	Belly-to-tendon ratio
Cadaver 1 - right (bilateral)	4.20	17.50	21.70	0.60	1:4.17
Cadaver 1 - left (bilateral)	4.40	17.80	22.20	0.65	1:4.05
Cadaver 2 - left (unilateral)	5.10	16.20	21.30	0.55	1:3.18
Mean ± SD	4.57 ± 0.46	17.17 ± 0.85	21.73 ± 0.45	0.60 ± 0.05	1:3.80

Data for each side are presented as raw measurements in centimeters, whereas pooled data are expressed as mean ± SD for the three identified psoas minor muscles (n = 3). The belly-to-tendon ratio is expressed as 1, where X = tendon length/belly length. The mean ratio (1:3.80) represents the arithmetic mean of the three individual ratios; when calculated from the mean belly and tendon lengths, the ratio is 1:3.76, with the tendon accounting for approximately 79% of the total muscle length. Because only three muscles were available for analysis, only descriptive statistics are reported, and no inferential statistical tests (e.g., a paired t-test for side-to-side comparisons or a one-sample t-test against published norms) were performed. Given the small sample size, the individual raw measurements are more informative than the pooled mean ± SD values, which should therefore be interpreted with caution.

The salient findings of the present cadaveric study are a very low overall observed frequency (6.67%) of the psoas minor in this single-institution cadaveric sample; no isolated right-sided muscle was identified (the positive sides comprised one bilateral pair and one left-sided muscle), although no directional left-versus-right inference can be drawn from only three positive muscles; consistent classical origin from T12-L1 and insertion into the pecten pubis and iliopubic eminence, with lateral blending into the iliac fascia; and a predominantly tendinous muscle with a small, slender belly.

## Discussion

The present observational cadaveric study, conducted on 30 adult human cadavers (60 sides) available at a single tertiary-care institution in Vadodara, Gujarat, documented the psoas minor muscle in two of 30 cadavers (2/30; 6.67%) and in three of 60 sides (3/60; 5.00%), with one bilateral and one unilateral left-sided presentation. This observed frequency is markedly lower than the classically reported prevalence of 40-50% [1,3,4]. It falls at the lower end of the wide global range reported, prompting a detailed comparison with the existing literature.

Cadaveric studies from different geographic regions have reported widely divergent prevalence rates. Farias et al. reported a frequency of 42.90% in a Brazilian series with a slight left-sided predominance [8]. Dragieva et al. reported a 60.00% prevalence in a Bulgarian cadaveric series and emphasized its surgical relevance [11]. Hanson et al. demonstrated a marked ethnic difference in the United States, identifying the muscle in 87.00% of white but only 9.00% of Black cadavers [12]. By contrast, studies from the Indian subcontinent have reported lower and more variable frequencies. Joshi et al., in a Western Indian sample, found the muscle in approximately 55.00% of specimens with considerable asymmetry [21]. Patil et al. reported a moderate frequency with notable morphometric variability in a North Indian cohort [9]. Kokati et al. specifically highlighted a comparatively lower frequency in a South Indian series and attributed the finding to probable ethnic and regional factors [10]. Our findings from this single institution in Vadodara, Gujarat, add to this body of evidence and are consistent with the view that the psoas minor may be less frequent in some Indian populations than in many Western ones.

Several explanations may account for the low frequency observed in the present study. Embryologically, the psoas minor develops from the hypaxial lumbar myotomes and, in quadrupedal mammals, serves as an important flexor of the trunk on the pelvis. With the evolutionary shift to bipedal locomotion in hominins, this role has been progressively assumed by the psoas major, rectus abdominis, and iliacus, and the psoas minor has therefore undergone regressive changes, persisting as a vestigial element whose presence shows substantial inter-individual variation [13,22]. Genetic, epigenetic, and possibly environmental factors acting during embryonic myogenesis may therefore determine whether the muscle persists in a given individual, explaining the inter-population variability observed [13,22]. Our observations in this small single-institution sample, together with those of Kokati et al. [10], are consistent with the possibility that the psoas minor may be less frequently present in some Indian populations; however, given our very small number of positive cases and single-center design, this should be regarded as hypothesis-generating only, and larger multicentric and molecular studies would be required to explore it.

The morphometric profiles of the three muscles examined in the present study were largely consistent with published data. The mean belly length of 4.57 cm and mean tendon length of 17.17 cm fall within the ranges reported by Patil et al. (belly 4.2-6.8 cm; tendon 15.1-19.6 cm) [9] and by Singh and co-workers (total length approximately 19-24 cm) [7]. The belly-to-tendon length ratio of approximately 1:3.80 likewise accords with the classical description of a short muscular belly paired with a disproportionately long tendon [1,3]. Protas et al., in a detailed cadaveric dissection, additionally observed occasional higher thoracic origins and double bellies [23], none of which were identified in the present series. The uniform morphological pattern observed here, classical T12-L1 origin, single belly, and standard insertion on the pecten pubis and iliopubic eminence, suggests that when the muscle is present in this population, it tends to conform to the orthodox anatomical description rather than exhibiting the aberrant patterns reported by Karrar [14] and Tubbs et al. [24].

The clinical relevance of the psoas minor extends across multiple specialties. During retroperitoneoscopic procedures such as donor nephrectomy, lymphadenectomy, and sympathectomy, an unexpected psoas minor may be misidentified as a neural or vascular structure, leading to unnecessary sacrifice or iatrogenic injury [15,16]. In anesthesia, the psoas compartment block relies on precise targeting of the psoas sheath, so variability in the psoas minor tendon is relevant during ultrasound-guided approaches. Radiologically, awareness of the morphological diversity of the psoas group on CT or MRI is important, as an underlying unilateral psoas minor variant may otherwise be misread as pathology [17,18]. From a physiotherapy perspective, the muscle has been implicated in lumbopelvic pain syndromes, anterior pelvic tilt, and gait disturbances [19]. Finally, atypical bilateral persistence or coexistence with accessorius muscles has been documented in cadaveric studies and should be sought during routine dissection [20].

The principal strengths of the present study include systematic bilateral dissection of each cadaver, application of a standardized measurement protocol using a digital caliper and non-elastic thread, and double verification of each measurement by the primary investigator and the guide. Further, the four-year duration across three consecutive MBBS batches enabled the accumulation of a homogeneous, Gujarat-based cadaveric cohort, adding region-specific data to an area where published evidence is sparse.

Before concluding the present study, the following limitations must be acknowledged. First, it was conducted at a single institution on a geographically restricted cohort, and the findings may not be directly generalizable to other regions or ethnic groups within India. Second, although 30 cadavers is a commonly used number for such studies, the low observed frequency produced a small absolute number of positive muscles (n = 3), limiting any inferential analysis of morphometric parameters. Third, formalin embalming is known to cause some tissue shrinkage that may influence absolute measurements [9,25]. Fourth, functional attributes such as contractile force and electromyographic activity cannot be assessed in cadaveric material. Fifth, histological and immunohistochemical characterization of the muscle fibers was not performed. Sixth, donor anthropometric data, including age, body height, and body weight, and the precise duration of formalin fixation, were not recorded because muscle length and diameter may be influenced by overall body size; size-adjusted analysis could not be undertaken, and the absolute morphometric values reported here must be interpreted with this limitation in mind. The demographic details (age, height, and body weight) of the two cadavers in which the psoas minor was identified were likewise not available. Finally, detailed ethnic, occupational, and clinical histories of the donors were unavailable, limiting deeper interpretation. Additionally, the cadaveric cohort was male-predominant (24 of 30; 80%), which limits assessment of any sex-related differences in the frequency or morphometry of the psoas minor; with only two positive cadavers, the exact binomial 95% confidence interval around the 6.67% frequency is wide (approximately 0.8-22.1%), so the point estimate is imprecise. Formal intra-observer and inter-observer reliability statistics (e.g., intraclass correlation coefficient) were not computed, given the very small number of positive muscles.

## Conclusions

The present cadaveric study demonstrated an overall frequency of the psoas minor of 6.67% in 30 adult human cadavers from a single tertiary-care institution in Vadodara, Gujarat, considerably lower than the classically reported prevalence of 40-50% and consistent with mounting evidence of ethnic and geographical variation. When present, the muscle conformed to the classical morphological pattern of a small, fusiform, single-bellied muscle arising from T12 and L1 and inserting, through a long, slender tendon, into the pecten pubis and iliopubic eminence and blending laterally with the iliac fascia. Awareness of this infrequent presence and of the morphometric profile detailed here is clinically relevant for retroperitoneal surgery, psoas compartment block, abdominopelvic imaging, and physiotherapeutic management of lumbopelvic dysfunction.

Larger multicentric cadaveric studies, combined with in vivo imaging-based surveys, are recommended to refine the regional epidemiology of this vestigial muscle in Indian populations and to clarify the genetic and embryological determinants of psoas minor agenesis.
